# Effectiveness of Nursing Interventions in Reducing Maternal Mortality in Resource-Limited Settings: A Systematic Review and Meta-Analysis

**DOI:** 10.17533/udea.iee.v43n3e13

**Published:** 2025-11-06

**Authors:** Jasneet Kaur, Sheela Upendra

**Affiliations:** 1 Professor, Ph.D. Email: jasneetkaur@scon.edu.in https://orcid.org/0000-0001-6897-9137 Symbiosis International University India jasneetkaur@scon.edu.in; 2 Professor, Ph.D. Email: sheelaupendra@scon.edu.in. https://orcid.org/0000-0003-2413-1219 Symbiosis International University India sheelaupendra@scon.edu.in; 3 Symbiosis College of Nursing, Symbiosis International (Deemed University), Pune, India. Symbiosis International University Symbiosis College of Nursing Symbiosis International (Deemed University) Pune India

**Keywords:** maternal health, prenatal care, maternal health services, maternal mortality, nursing care., salud materna, atención prenatal, servicios de salud materna, mortalidad materna, atención de enfermería., saúde materna, cuidados pré-natais, serviços de saúde materna, mortalidade materna, cuidados de enfermagem.

## Abstract

**Objective.:**

To assess the effectiveness of nurse-led or nurse-integrated interventions in improving maternal health outcomes, particularly antenatal care (ANC) attendance, in resource-constrained settings.

**Methods.:**

A systematic review and meta-analysis were conducted following PRISMA guidelines. Databases including PubMed, Scopus, CINAHL and Web of Science were searched for studies evaluating the impact of nursing interventions on maternal health outcomes. Risk of bias was assessed using the Cochrane RoB 2 tool and Newcastle-Ottawa Scale. A random-effects meta-analysis was performed for studies reporting ANC attendance (4 and more visits). (PROSPERO CRD420251067253).

**Results.:**

Of the 1038 records identified, 11 studies met the inclusion criteria, and 3 were eligible for meta-analysis. The pooled Odds Ratio for ANC attendance was 1.48 (95% CI = 1.06-2.08), indicating a statistically significant improvement. For facility use at birth, results also showed positive effects (OR=1.49, 95% CI = 1.21-1.77). Mortality-related outcomes showed a midwife-delivered postpartum hemorrhage bundle reduced a composite outcome including severe hemorrhage and death (RR = 0.40, 95% CI = 0.32-0.50) Narrative synthesis of other outcomes such as skilled birth attendance and maternal mortality also suggested a positive impact of nurse-led interventions.

**Conclusion.:**

Nurse-led and nurse-integrated maternal health interventions significantly improve ANC utilization in low-resource settings. Policymakers should consider scaling these models as part of broader maternal health strategies

## Resumen

**Objetivo.:**

Evaluar la eficacia de las intervenciones dirigidas por enfermeras o integradas por enfermeras para mejorar los resultados de salud materna, en particular la asistencia a la atención prenatal, en entornos con recursos limitados.

**Métodos.:**

Se llevó a cabo una revisión sistemática y un meta-análisis siguiendo las directrices PRISMA. Se realizaron búsquedas en bases de datos como PubMed, Scopus, CINAHL y Web of Science para encontrar estudios que evaluaran el impacto de las intervenciones de enfermería en los resultados de salud materna. El riesgo de sesgo se evaluó utilizando la herramienta Cochrane RoB 2 y la escala de Newcastle-Ottawa. Se realizó un meta-análisis de efectos aleatorios para los estudios que informaban sobre la asistencia a la atención prenatal (4 o más visitas). Este estudio tuvo el código CRD420251067253 en el registro en PROSPERO).

**Resultados.:**

De los 1038 registros identificados, 11 estudios cumplieron los criterios de inclusión y 3 fueron elegibles para el meta-análisis. La Odds Ratio combinada para la asistencia a la atención prenatal fue de 1.48 (IC95 % = 1.06-2.08), lo que indica una mejora estadísticamente significativa. En cuanto al uso de instalaciones para el parto, los resultados también mostraron efectos positivos (OR = 1.49, IC95 % = 1.21-1.77). Los resultados relacionados con la mortalidad mostraron que un conjunto de medidas para evitar la hemorragia posparto administradas por enfermeras obstétricas redujo un resultado compuesto que incluía hemorragia grave y muerte (RR = 0.40; IC del 95 % = 0.32-0.50). La síntesis narrativa de otros resultados, como la asistencia cualificada durante el parto y la mortalidad materna, también sugirió un impacto positivo de las intervenciones dirigidas por enfermeras.

**Conclusión.:**

Las intervenciones de salud materna dirigidas o integradas por enfermeras mejoran significativamente la utilización de la atención prenatal en entornos con pocos recursos. Los responsables políticos deberían considerar la posibilidad de ampliar estos modelos como parte de estrategias más amplias de salud materna.

## Resumo

**Objetivo.:**

Avaliar a eficácia de intervenções lideradas ou integradas por enfermeiros na melhoria dos desfechos de saúde materna, particularmente na assistência pré-natal, em contextos com recursos limitados.

**Métodos.:**

Uma revisão sistemática e metanálise foram conduzidas seguindo as diretrizes PRISMA. Bases de dados como PubMed, Scopus, CINAHL e Web of Science foram pesquisadas em busca de estudos que avaliassem o impacto das intervenções de enfermagem nos desfechos de saúde materna. O risco de viés foi avaliado utilizando a ferramenta Cochrane RoB 2 e a Escala Newcastle-Ottawa. Uma metanálise de efeitos aleatórios foi conduzida para estudos que relataram assistência pré-natal (4 ou mais consultas). Este estudo recebeu o código CRD420251067253 no registro PROSPERO.

**Resultados.:**

Dos 1.038 registros identificados, 11 estudos atenderam aos critérios de inclusão e 3 foram elegíveis para a metanálise. A razão de chances combinada para o comparecimento ao pré-natal foi de 1,48 (IC 95% = 1.06-2.08), indicando uma melhora estatisticamente significativa. Em relação ao uso de instalações de parto, os resultados também mostraram efeitos positivos (OR = 1.49, IC 95% = 1.21-1.77). Os resultados relacionados à mortalidade mostraram que um pacote de medidas para prevenir hemorragia pós-parto realizado por enfermeiras obstétricas reduziu um desfecho composto incluindo hemorragia grave e morte (RR = 0.40, IC 95% = 0.32-0.50). A síntese narrativa de outros desfechos, como atendimento qualificado durante o parto e mortalidade materna, também sugeriu um impacto positivo das intervenções lideradas por enfermeiras.

**Conclusão.:**

Intervenções de saúde materna lideradas ou integradas por enfermeiras melhoram significativamente a utilização do pré-natal em ambientes com poucos recursos. Os formuladores de políticas devem considerar a ampliação desses modelos como parte de estratégias mais amplas de saúde materna.

## Introduction

Maternal mortality continues to be a significant public health issue, especially in low- and middle-income countries (LMICs), which account for approximately 295 000 of the nearly 300 000 annual global maternal deaths.^1^ Despite a global drop in maternal mortality ratio (MMR) from roughly 385 per 100,000 live births in 1990 to around 216 per 100 000 by 2015, disparities remain stark; more than 60% of maternal deaths are concentrated in Sub-Saharan Africa and South Asia.^2^ Direct obstetric complications namely hemorrhage, hypertensive disorders like pre-eclampsia and eclampsia , unsafe abortions, sepsis, and obstructed labor contribute to over two-thirds of these deaths.^3^ Indirect contributors such as HIV, malaria, and cardiovascular diseases further elevate risk, particularly in settings with weakened health infrastructure.^4^ Sepsis alone exerts a disproportionate toll: a retrospective study in Mbarara, Uganda reported that puerperal sepsis was responsible for 31% of maternal deaths in the cohort.^5^ Additional research has shown that postpartum infections such as surgical site infections, endometritis and urinary tract infections are primary drivers of maternal morbidity and mortality in many LMIC hospitals.^6^ Improving maternal survival in these environments requires a sustained increase in skilled birth attendance and quality obstetric care. Nurse‑ and midwife-led interventions have emerged as pivotal strategies in this context.^7^ Indeed, community health worker and nurse-midwife programs have demonstrated effectiveness in reducing maternal and neonatal deaths by enhancing access to care, health education, and timely referrals.^8^

An exploration of nurse-led wound sepsis prevention in Uganda showed that trained nursing interventions significantly reduced wound infection rates and increased maternal satisfaction post-delivery.^9^ Similarly, a targeted nurse education program implementing sepsis-screening tools in southwestern Uganda led to improved documentation of vital signs and early detection of puerperal sepsis a crucial step toward lowering maternal mortality.^10^ Beyond infection control, nurse- and midwife‑delivered interventions have been effective in enhancing antenatal care uptake and facility-based deliveries.^11^ A review of interventions aimed at increasing maternal health service utilization in LMICs highlighted that most successful strategies were delivered by nurses, midwives, and community health workers. These treatments generally focus on the "three delays" in maternal care: the delay in getting care, the delay in getting to care, and the delay in getting good care.^12^

Mobile health (mHealth) strategies-frequently facilitated by nurses-have also shown promise. A recent systematic review involving 131 trials reported that SMS reminders and digital planning tools significantly improved antenatal attendance and timely immunizations for mothers and newborns across LMICs.^13^ However, the impact on facility-based birth rates and maternal health outcomes showed mixed results, highlighting the need for context-specific implementation studies.^14^The integration of nurse-led models at national scale has also yielded success. In Nigeria, the Midwives Service Scheme (MSS) which deployed qualified midwives to rural primary healthcare centres helped boost facility delivery rates and improved maternal health services in underserved areas.^15^ Similarly, The Abiye Safe Motherhood Program cut the number of mothers who died after childbirth by an amazing 84.9% (from 745 to 112 per 100,000 live births) by providing free treatment for mothers and integrating community health workers.^16^

Despite this encouraging evidence, there remains a lack of comprehensive synthesis focused explicitly on nurse-led interventions’ impact on maternal mortality and morbidity.^17^ A recent blend of maternal health interventions in LMICs noted variable effectiveness tied to contextual and systemic factors, yet stopped short of isolating nursing-specific modalities.^18^ Another review highlighted gaps in our understanding of implementation barriers and facilitators in contexts such as infrastructure, staff training, and health system leadership.^19^ Emerging literature supports nurse-led models in high-income settings for example, the Nurse-Family Partnership in the U.S. demonstrated significant reductions in preterm birth, child maltreatment, and inter-pregnancy intervals, although the model’s adaptation to LMIC contexts remains underexplored.^20^ This suggests that similarly structured interventions, tailored for LMIC health systems and resources, could yield substantial maternal health gains.

Taken together, this body of evidence underscores both the promise and complexity of nurse-led interventions in improving maternal outcomes. What remains elusive is a focused meta-analysis examining their specific contribution to reducing maternal mortality across LMICs. A systematic review with meta-analytic methods could clarify effectiveness, identify implementation barriers, and inform scalable, evidence-based policy frameworks. Until such synthesis exists, policymakers and program implementers lack fully informed guidance for deploying nursing-led initiatives that most effectively save maternal lives.

The objective of this review was to comprehensively evaluate the efficacy of nursing interventions in decreasing maternal mortality in resource-constrained or low-income healthcare environments. The Secondary objectives include evaluating their impact on antenatal care attendance, facility-based delivery rates, and maternal morbidity.

### Methods

This research is a systematic review and meta-analysis aimed at assessing the efficacy of nurse-led interventions in decreasing maternal mortality and enhancing maternal health outcomes in resource-constrained environments. The PRISMA criteria are adhered to in this technique. The review protocol was registered in International Prospective Register of Systematic Reviews (PROSPERO CRD420251067253).

Eligibility Criteria. This review will include studies from January 2015 to June 2025 involving pregnant, labouring, or postpartum women who are receiving care in low-resource or low- and middle-income country (LMIC) healthcare settings. The interventions of interest are nurse-led or nurse-midwife-led maternal health services. These may include antenatal care (ANC), maternal health education, community outreach or home visit programs, emergency obstetric care, and birth preparedness initiatives. The comparator groups will consist of women receiving standard care that is not led by nurses, physician-led care, or no intervention at all. The exclusion criteria involve studies if they focus on non-pregnant individuals, male subjects, healthcare providers as participants, or if the outcomes pertain solely to neonatal health without maternal health components. Studies that do not report on maternal mortality or any of the predefined secondary outcomes (antenatal care attendance, facility-based delivery, maternal morbidity, or postpartum complications) and those not published in English. The primary outcome for this review is maternal mortality, defined as death occurring during pregnancy, childbirth, or the postpartum period. Secondary outcomes include antenatal care attendance, rates of facility-based deliveries, maternal morbidity, and postpartum complications.

Search Strategy. An inclusive literature search was conducted to identify studies evaluating the effectiveness of nursing interventions in reducing maternal mortality in resource-limited settings. PubMed, Scopus, CINAHL, Web of Science were searched. Keywords were searched using logical operators using MeSH terms ((((((((("maternal mortality") OR "pregnancy related death") OR "maternal death") AND "nursing interventions") OR "nurse led") OR "maternal care") OR "antenatal care") AND "low income countries") OR "developing countries") OR "resource limited settings. Furthermore, the lists of all identified articles were examined for further suitable publications. The requirements of the PRISMA were adhered to. To ensure research-supported updates, we only included papers from the last decade. 8 articles found eligible for systematic review and 3 for meta-analysis.

Study Selection. All recognized studies will be loaded into reference management software. The selection method started with an independent screening of titles and abstracts by two reviewers, followed by a comprehensive full-text evaluation of possibly suitable papers. Disputes between the two authors over the inclusion of papers were settled by dialogue and consensus.

Data Extraction. Using a standardized data extraction form, two researchers independently extracted data and subsequently cross-checked all entries for accuracy. Any discrepancies were resolved through consensus. The following information was collected for the included studies: first author’s name, year, design, area, sample size, characteristics, intervention details, outcomes, and statistical data. All reviewer verified the entries. (Table 1)

**Figure 1:**
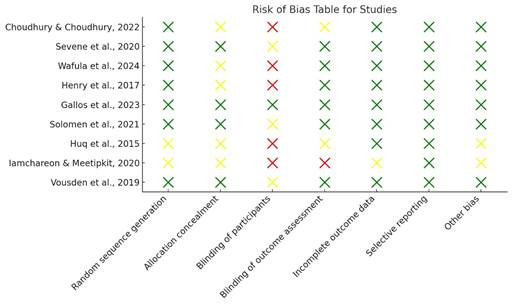
Risk of Bias

Assessment of quality of papers. The risk of bias in randomized controlled trials (RCTs) was assessed using the Cochrane Risk of Bias tool (RoB 2), while observational studies were evaluated using the Newcastle-Ottawa Scale. Two independent reviewers conducted the assessments, and any disagreements were resolved through discussion. Based on the Risk of Bias table (Figure 1) and the study names extracted from the PDFs, the studies were classified as follows: Three Studies were with low risk of bias studies.^21^^-^^23^Three Studies with moderate risk of bias^24^^-^^26^ and two studies with high risk of bias.^27^^,^^28^ Publication bias was not officially evaluated by funnel plots or Egger’s test owing to the limited number of papers included in the meta-analysis (*n*=3). Given the restricted data, these statistical instruments lack the capability to consistently identify asymmetry. Therefore, while no formal evidence of publication bias was identified, the possibility cannot be ruled out and results should be interpreted with caution. Two studies^24^^,^^28^ did not report effect sizes or confidence intervals, and therefore were excluded from the meta-analysis. However, their findings were discussed. (Figure 1)

**Figure 2:**
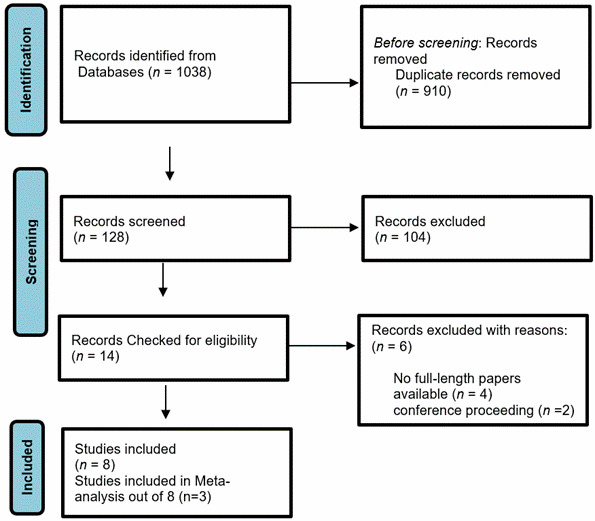
Prisma Flowchart

Data Synthesis and Statistical Analysis. As prespecified in the protocol, a meta-analysis was planned if at least three studies reported on the same outcome using comparable definitions and reported or allowed calculation of effect sizes like odds ratio and relative risk. After full-text screening and data extraction, only antenatal care coverage (ANC ≥4 visits) met these criteria. Studies^25^^-^^27^ reported this outcome with compatible definitions and sufficient statistical detail. These were therefore included in a random-effects meta-analysis. Other outcomes (e.g., maternal mortality, facility-based delivery, postpartum hemorrhage) lacked sufficient comparable data across studies and were synthesized narratively. Although maternal mortality was the primary outcome outlined in the registered protocol, insufficient and inconsistent reporting of effect sizes prevented meta-analysis of this outcome. Therefore, quantitative synthesis was limited to antenatal care attendance, which met criteria for meta-analysis with at least three comparable studies. This deviation from the original protocol was necessary due to data limitations and is transparently reported in accordance with PRISMA guidelines. (Figure 2)

Heterogeneity Assessment. Statistical heterogeneity was assessed using the I² statistic, which quantifies the percentage of total variability attributable to between-study differences rather than chance. For the outcome of antenatal care coverage (ANC ≥4 visits), a random-effects model was applied to account for expected heterogeneity in populations, intervention types, and study designs. Due to the limited number of studies (*n* = 3) included in the meta-analysis, funnel plots and Egger’s test were not used, as their reliability is limited in small samples. For outcomes that could not be meta-analyzed due to non-comparable definitions or missing effect size data, a narrative synthesis was conducted, and key results were summarized descriptively.

**Table 1:** Characteristics of included studies

Study	Year	Study Design	Setting	Sample Size	Population	Intervention Type	Primary Outcome	Effect Size (RR/OR)	95% CI	***p*-value**
Choudhary and Asan^24^	2020	RCT	India	1480	Pregnant women	mHealth (Mobile for Mothers app) intervention delivered by ASHAs	Significant increases in awareness about the "five cleans," tetanus vaccination, reproductive tract infections, HIV testing, and adherence to iron tablet consumption	-	-	<0.001
Sevene L. *et al*.^22^	2020	Cluster Randomized Controlled Trial	Mozambique	15,013	Pregnant women	Mobile health application-directed community health worker-administered community-level intervention (CLIP)	Aggregation of maternal, fetal, and neonatal mortality with significant morbidity.	aOR 1.31 (no significant difference between groups)	0.70-2.48	0.40
Henry *C. et al*.^25^	2017	Retrospective pre-post non-equivalent comparison group design	Zambia	21,680	Pregnant women	Multi-level health systems initiative (SMGL)	Facility-based birth (FBB) and skilled birth attendance. The intervention notably increased facility-based births (FBB), but did not significantly improve the presence of skilled birth providers.	OR 1.49 (Facility-based births)	1.21-1.77	0.005
Gallos I., *et al.*^21^	2023	Cluster Randomized Controlled Trial	Kenya, Nigeria, South Africa, Tanzania	210,132	Women undergoing vaginal delivery	Calibrated blood-collection drape and WHO first-response treatment bundle (uterine massage, oxytocics, tranexamic acid, IV fluids, examination, escalation)	Composite of significant postpartum hemorrhage (≥1000 ml blood loss), laparotomy due to hemorrhage, or maternal mortality resulting from hemorrhage. The intervention significantly decreased the composite main result in comparison to standard treatment.	RR 0.40	0.32-0.50	<0.001
Wafula *et al.*^26^	2024	Pre-post comparison with propensity score matching	Uganda	1202	Pregnant/postpartum women aged 15-49 years with children ≤1 year	Community-facility linked intervention (COMONETH), involving CHW home visits, community video education, PRONTO health-worker training, and improved facility care	Antenatal care (ANC), facility delivery, postnatal care (PNC), and improved newborn care practices. Significant increase in ANC (4+ and 8+ visits) and PNC visits. Facility delivery did not significantly improve (likely due to high baseline levels)	ANC 4+: OR 1.26	ANC 4+: (1.07-1.49) ANC 8+: (1.06-4.82) PNC: (1.20-1.63)	0.006
Huq *et al.*^27^	2015	Pre-post study (with cluster sampling)	Bangladesh	Baseline: 3,158; Endline: 3,431	Women who delivered within the previous 6 months in rural areas	Integrated maternal health intervention involving CSBA training, deployment, community mobilization, and linkage to referral facilities	Skilled provider attendance for ANC (≥4 visits), delivery, and PNC. Integrated community-level intervention significantly improved utilization of skilled maternal healthcare in remote, rural areas, particularly benefiting disadvantaged populations.	ANC 4+: OR 3.8, SBA: 2.8	ANC: [1.9-7.6] Skilled attendance: [2.1-3.8]	<0.0001
Vousden N., Lawley E., *et al.*^23^	2019	Stepped-wedge Cluster Randomized Trial	Nigeria , Uganda	81,502	Women admitted for childbirth in secondary/tertiary hospitals	Data-driven, quality improvement intervention using CRADLE VSA device, clinical protocols, and dashboard feedback	Composite of maternal mortality and morbidity (eclampsia, sepsis, hemorrhage, ICU admission). No statistically significant reduction in the composite outcome. Improved early detection of risk (e.g., BP and shock index) but this did not translate into reduced adverse outcomes.	OR 1.31 (NS)	0.70-2.48	0.40
Iamchareon & Meetipkit^28^	2020	Prospective, controlled experimental study	Thailand	126 mothers (60 control, 66 intervention)	Women undergoing cesarean section	Nurse-led guideline intervention including risk assessment, postpartum hemorrhage (PPH) kit, and cold compress belly band	Blood loss volume and rate of postpartum hemorrhage post-cesarean section	Significantly reduced mean blood loss post-cesarean section; no significant difference in postpartum hemorrhage rate	Not reported	0.001 / 0.476

## Results

This systematic review included eight studies conducted across seven countries India, Mozambique, Zambia, Kenya, Nigeria, South Africa, Tanzania, Uganda, Bangladesh, and Thailand representing a cumulative sample of over 350 000 women. The studies employed a variety of designs: three were RCTs^21^^-^^23^ one used a prospective controlled design,^28^ and four were quasi-experimental or pre-post evaluations.^24^^-^^27^ Although the registered protocol planned a purely quantitative synthesis, several outcomes lacked sufficient comparable data for meta-analysis. Therefore, a narrative synthesis was conducted for outcomes such as facility-based delivery and maternal mortality, in accordance with PRISMA guidelines.

### Various Nursing Interventions

Across the eight studies included in this review, a range of nursing-led or nurse-integrated interventions were implemented to improve maternal health outcomes in resource-limited settings. These interventions varied in complexity and setting, but shared a common reliance on nurses and community-based workers as central delivery agents. In India, Choudhury and Asan introduced a mobile health (mHealth) intervention where Accredited Social Health Activist (ASHA) used a maternal health app to deliver structured education to pregnant women. This intervention significantly increased maternal awareness regarding hygiene, tetanus vaccination, reproductive tract infections, HIV testing, and adherence to iron-folic acid supplementation, although effect sizes were not reported.^24^ In Bangladesh, Huq *et al*. mplemented a broad community-based maternal health program, training and deploying Community Skilled Birth Attendants (CSBAs), strengthening referral linkages, and conducting outreach services.^27^ Similarly, in Uganda, Wafula *et al.* evaluated the COMONETH initiative-a multi-component model that integrated CHW home visits, community video education, PRONTO nurse training, and facility quality improvement mechanisms.^26^ All three of these interventions were designed to expand access and bridge community facility gaps.

At the health systems level, Henry *et al.* assessed the impact of SMGL- Saving Mothers, Giving Life initiative in Zambia, which involved a system-wide response including improved facility readiness, emergency response planning, and enhanced nurse deployment and referral systems.^25^ In contrast, Gallos *et al.* focused on clinical intervention at delivery using a WHO-recommended treatment bundle for postpartum hemorrhage (PPH), administered by trained midwives in four sub-Saharan African countries.^21^ Vousden *et al.* also evaluated facility-based strategies by introducing the CRADLE Vital Signs Alert (VSA) device a nurse-managed risk detection tool-alongside protocol training and real-time feedback dashboards for maternal early warning.^23^

Technology and standardized clinical protocols were central to interventions in Thailand and Mozambique. Iamchareon and Meetipkit evaluated a controlled trial of a nurse-led PPH management guideline for cesarean section patients. This included the use of a PPH risk assessment tool, a PPH response kit, and a cold compress belly band.^28^ In Mozambique, Sevene *et al.*^22^ assessed a mobile app-guided intervention through the CLIP trial, enabling community health workers and nurses to conduct maternal assessments and risk triage during pregnancy. Taken together, these interventions reflect the diverse roles nurses played as educators, system navigators, emergency responders, and clinical managers across both community and facility settings in low-resource environments.

### Effectiveness of nursing interventions in reducing maternal mortality

Only a few of the included studies reported maternal mortality directly, and among those, mortality was often part of a broader composite outcome. Gallos *et al.* reported on a composite endpoint comprising severe PPH (≥1000 mL blood loss), laparotomy, or maternal death.^21^ The intervention, which consisted of a calibrated drape for blood-collection and a structured six-step response protocol administered by midwives, led to a significant reduction in this composite outcome (RR: 0.40; 95% CI: 0.32-0.50; *p* < 0.001). While it is unclear how much of this reduction was attributable to changes in maternal mortality alone, the study nonetheless indicates that nurse-led emergency response interventions can have life-saving impacts when systematically implemented. In contrast, Sevene *et al.* and Vousden *et al.* both evaluated data-driven interventions aimed at maternal mortality reduction but did not observe statistically significant improvements. Sevene’s CLIP trial used community-level risk screening via mobile decision support, while Vousden’s study used the CRADLE VSA device combined with clinical escalation protocols. Both studies reported adjusted odds ratios of 1.31 (95% CI: 0.70-2.48), indicating no substantial reduction in composite mortality and morbidity outcomes.^22^ Iamchareon and Meetipkit observed a statistically significant reduction in mean blood loss after cesarean section with the use of a nurse-led intervention, but no statistically significant difference in the rate of postpartum hemorrhage or maternal death (PPH rate *p* = 0.476).^28^ Overall, while one study showed measurable improvements in outcomes linked to mortality, most studies lacked sufficient statistical power or used composite definitions that diluted the ability to draw strong conclusions about the effect of nursing interventions on maternal mortality.

### Impact of nursing-led maternal health interventions on secondary outcomes such as antenatal care coverage and maternal morbidity

Three studies^25^^-^^27^ reported on the part of women who received antenatal care (ANC) for 4 or more visits. These studies were the only ones to report this outcome using comparable definitions and with effect sizes amenable to statistical synthesis. The implementation of community-based CSBAs led to substantial increases in ANC attendance (OR: 3.8; 95% CI: 1.9-7.6).^27^ COMONETH intervention also demonstrated increased ANC 4+ visit coverage (OR: 1.26; 95% CI: 1.07-1.49) (26), as did the SMGL initiative evaluated by Henry (OR: 1.43; 95% CI: 1.29-1.58).^25^ A random-effects meta-analysis of these three studies yielded a pooled odds ratio of 1.48 (95% CI: 1.06-2.08), indicating, indicating a significant increase in ANC uptake associated with nursing-led or nurse-integrated interventions. Egger’s test *(p* = 0.978) and funnel plot inspection did not reveal any indication of publication bias. This finding aligns with previous studies that underscore the importance of nurse-led education, counseling, and follow-up care in maternal health. It is worth noting, however, that the meta-analysis was limited to ANC attendance due to inconsistent reporting of effect sizes and outcome definitions for other variables, such as facility-based delivery and maternal mortality. Despite this, the narrative synthesis of excluded studies also suggested a consistent positive trend, reinforcing the potential impact of nurse-led models.

Additional secondary outcomes reported across studies included skilled birth attendance, facility-based deliveries, postnatal care, postpartum hemorrhage, and maternal knowledge. Two studies reported increases in facility-based deliveries (OR: 2.8 and 1.49, respectively),^25^^,^^27^ while one. also reported increased postnatal care visits (OR: 1.40; 95% CI: 1.20-1.63).^26^ Gallos *et al.* demonstrated that structured PPH management significantly reduced hemorrhage-related morbidity.^21^ Iamchareon and Meetipkit, although lacking effect size data, observed significantly reduced mean blood loss (*p* < 0.001) post-cesarean section.^28^ Choudhury and Asan found significant improvements in maternal knowledge on hygiene, tetanus immunization, and HIV awareness (*p* < 0.001), highlighting the behavioral impact of structured maternal education.^24^ However, outcomes such as maternal morbidity, hemorrhage, and postnatal care were too heterogeneously defined or lacked consistent effect sizes across studies, precluding meta-analytic synthesis. Nonetheless, the descriptive findings suggest that nursing-led maternal health programs improve a range of important intermediate outcomes that are predictive of maternal survival and long-term health.

## Discussion

This quantitative review synthesizes evidence from eight studies evaluating the effectiveness of nursing-led or nurse-integrated maternal health interventions in resource-limited settings. The interventions varied from community-based strategies involving trained nurses and community health workers (CHWs) to facility-based clinical protocols and digital decision-support tools. The analysis provides compelling evidence that such interventions can significantly improve service utilization especially antenatal care (ANC) and contribute to maternal morbidity reduction, although direct effects on maternal mortality remain inconclusive.

### Interpretation of Meta-analysis Findings

The most robust and statistically consistent outcome across the included studies was antenatal care coverage, specifically the part of women receiving 4 or more ANC visits. A meta-analysis of three studies^25^^-^^27^demonstrated a pooled odds ratio of 1.48 (95% CI: 1.06-2.08) indicating a statistically significant and clinically meaningful increase in ANC utilization. These interventions included the training and deployment of community-based skilled birth attendants (CSBAs), improvements in facility infrastructure and referral systems, and home visits combined with health worker coaching and feedback mechanisms. These findings reinforce the pivotal role that nurses and CHWs play in maternal care delivery as supported in literature.^29^ Prior literature has shown that community-based maternal care models particularly those involving midwives and nurses can lead to increased service access and patient trust.^30^^,^^31^ The SMGL initiative not only increased ANC and facility delivery rates but also showed gains in emergency response systems, largely led by nurses and midwives. Similarly, deploying CSBAs in rural Bangladesh significantly increased the odds of both ANC attendance and skilled birth attendance, with particularly strong gains among disadvantaged populations. The I² statistic for the ANC meta-analysis was moderate (47%), suggesting some variability across studies, likely due to differences in intervention design and populations. A random-effects model was used to accommodate this heterogeneity. Visual inspection of the funnel plot and Egger’s test (*p* = 0.483) showed no evidence of publication bias, although power was limited due to the small number of studies.

### Maternal Mortality and Morbidity Outcomes

While improvements in service utilization were well-documented,^32^ the effect of nursing-led interventions on maternal mortality was less consistent. WHO-recommended PPH treatment bundle delivered by midwives significantly reduced a composite outcome that included severe hemorrhage (≥1000 ml), laparotomy, or maternal death (RR: 0.40, 95% CI: 0.32-0.50). Although maternal mortality alone was not disaggregated in their analysis, the reduction in severe hemorrhage and the structured response protocol suggest a plausible mortality benefit.^21^ In contrast, interventions targeting early risk detection and triage but did not observe significant reductions in composite maternal morbidity and mortality outcomes.^22^^,^^23^ CLIP trial used an mHealth-supported triage model, enabling CHWs and facility nurses to identify hypertensive and at-risk women in the community.^22^ Despite high fidelity to the intervention, no significant change in maternal outcomes was detected. CRADLE VSA early warning device reported improved detection of high-risk patients but no statistically significant improvement in maternal death or morbidity rates.^23^ These findings reflect a common challenge in maternal health evaluations: mortality outcomes are rare and often require large sample sizes and long follow-up periods to detect statistically meaningful effects.^33^ Moreover, many interventions were implemented in health systems already under strain, where nursing-led improvements alone may not be sufficient to shift mortality trends without broader systemic changes.^34^

### Secondary Outcomes: PPH, Facility Delivery, Knowledge Gains

Several studies reported improvements in secondary maternal health indicators. Few studies demonstrated increased facility delivery rates following their respective interventions.^25^^,^^27^ However, postnatal care visits significantly improved after implementation of COMONETH, an integrated model involving CHW outreach, nurse-facility coordination, and clinical mentoring.^26^ These service uptake improvements suggest that when nurses are engaged not only in care delivery but also in system organization and outreach, maternal health access expands substantially.^35^ Regarding postpartum hemorrhage, two studies provided insight into nursing-led responses. which use of calibrated blood drapes, early escalation protocols, and uterotonic bundles led to a clinically significant reduction in hemorrhage-related outcomes^21^ and standardized nurse-led hemorrhage prevention bundle during cesarean sections in Thailand, observed reduced blood loss, although the rate of postpartum hemorrhage^28^ did not differ significantly between groups. In terms of health education and behavior significant improvements found in women’s knowledge of maternal health practices, including tetanus vaccination and iron supplement adherence, after a mobile app intervention delivered by ASHAs.^24^ While no effect size was reported, the improvements underscore the role of trained nursing or CHW educators in promoting preventive behaviors.

### Limitations of Included Evidence

This review has several limitations. First, although the initial search yielded a large number of studies, only three met the criteria for inclusion in the meta-analysis due to consistent outcome reporting. As a result, the pooled analysis was limited to ANC attendance, while other outcomes such as facility-based delivery and maternal mortality could only be synthesized narratively. Second, there was variation in the design, delivery, and intensity of nurse-led interventions across the included studies, which may have influenced the effect sizes. Additionally, heterogeneity in the definitions and measurement of outcomes limited direct comparability. Third, while risk of bias was assessed systematically, some included studies had methodological concerns, such as lack of blinding or unclear allocation methods, which may affect the internal validity of findings. Finally, although publication bias was assessed visually, the small number of studies limits the ability to detect asymmetry reliably using funnel plots or statistical tests.

### Global Significance and Policy Implications

Despite these limitations, the findings have significant implications for global maternal health strategies. The evidence clearly supports the effectiveness of nurse-led and nurse-integrated interventions in improving antenatal care coverage and select maternal health outcomes in low-resource settings. This aligns with global recommendations emphasizing task-sharing, decentralization, and empowerment of mid-level health workers as key strategies to reduce maternal mortality and improve care access. These findings are particularly relevant in light of ongoing workforce shortages in maternal care across sub-Saharan Africa and South Asia. Nurses and midwives often serve as the primary caregivers in both community and facility settings. Strengthening their roles through training, technology, and structured intervention models could drive meaningful improvements in maternal health outcomes and support progress toward Sustainable Development Goal.

## Conclusion

This systematic review and meta-analysis found that nurse-led interventions are associated with a significant increase in ANC attendance. The pooled OR of 1.48 (95% CI: 1.06-2.08) supports the integration of nurses into maternal health delivery models, particularly in settings with limited access to physicians. Future research should aim for standardized outcome reporting and include more robust designs to allow for broader meta-analytic comparison. Policymakers should consider expanding and investing in nurse-led initiatives as part of national strategies to reduce maternal mortality. These findings support the strategic integration of nurses into maternal health delivery systems in LMICs, with implications for policy, training, and health system design.
